# Smartphone-Based Postoperative Wound Assessment Following Laparoscopic Surgery in a Resource-Limited Setting: A Prospective Cohort Study

**DOI:** 10.3390/bioengineering13060663

**Published:** 2026-06-05

**Authors:** Marryam Riaz Farooqui, Hamza Waqar Bhatti, Muhammad Umar Javed, Aurangzeb Khan, Muhammad Hanif

**Affiliations:** 1 Transplant and Renal Surgery, Guy’s & St. Thomas NHS Foundation Trust, London SE1 9RT, UK; 2 Department of Surgery and Cancer, Imperial College London, London W12 OHS, UK; 3 General Surgery, Surgical Unit-II, Benazir Bhutto Hospital, Rawalpindi 46000, Pakistansurgeonhanif@yahoo.com (M.H.)

**Keywords:** telemedicine, wound, smartphone, general surgery, bariatric surgery, laparoscopy

## Abstract

Remote postoperative wound assessment may help improve follow-up after laparoscopic surgery in resource-limited settings. This study evaluated the feasibility and patient satisfaction of smartphone-based postoperative wound assessment following general and bariatric laparoscopic surgery. We conducted a prospective cohort study from June 2022 to June 2023 at a public sector teaching hospital. Consecutive adult patients undergoing elective laparoscopic general or bariatric procedures were invited to participate. Consenting patients submitted wound photographs and clinical queries to their surgeon within 14 days of discharge using an encrypted messaging platform. The primary outcome was patient satisfaction measured using the Patient Satisfaction Questionnaire Short Form (PSQ-18). Secondary outcomes included the proportion of patients requiring escalation to in-person review and the type of remote intervention provided. A total of 113 patients were enrolled. Of these, 21 (18.6%) required escalation to in-person review. Among the 92 patients managed remotely, 52 (46.0%) received reassurance only and 40 (35.4%) required medication prescription or adjustment. The mean PSQ-18 score for the cohort was 79.66 ± 11.24 (range 18–90). Satisfaction was comparable across procedure types. Smartphone-based postoperative wound assessment appears feasible and acceptable in this setting, with most postoperative concerns managed remotely and favourable patient satisfaction. Further controlled studies are needed to assess safety, diagnostic accuracy, and cost-effectiveness.

## 1. Introduction

Postoperative surgical site infection (SSI) remains a major cause of morbidity, delayed recovery, readmission, and increased healthcare use after surgery [1,2,3,4]. As more patients are discharged earlier and recover outside the hospital [5], remote postoperative monitoring has become increasingly important. Recent multimodal machine-learning work has shown that wound images and patient-reported outcome measures can be combined to predict confirmed SSI within 48 h, with performance comparable to clinician triage and a simulated 80% reduction in staff time, supporting the value of digital surveillance in surgical care [6].

Remote wound monitoring has also been shown to work in practical service models [7,8,9]. In Singapore, an inaugural tele-wound monitoring service for acute wounds was described in 204 primary care patients, and the service was reported as a valuable alternative to face-to-face wound care; age and healing duration predicted utilization [10]. Smartphone-based wound follow-up has also been explored in hospital practice. In an Italian experience during the pandemic period, wound recovery was monitored with a smartphone, with 255 total consultations, a significant reduction in outpatient visits, and high patient satisfaction; the authors concluded that monitoring wound recovery in this way was very easy [11]. This supports the idea that mobile messaging may be a practical option for postoperative wound surveillance when patients already use smartphones and dedicated digital platforms are not available [12,13].

More recent studies have focused on refining wound-image assessment using digital platforms and artificial intelligence. The WISDOM AI study developed a smartphone-based wound imaging platform to prioritise surgical wound images for clinician review and reported 89% sensitivity for priority review together with perfect intra-rater reliability [14]. In a paired cohort study, an AI-based wound monitoring application achieved 100% sensitivity, 83.13% specificity, and substantial agreement with human observers (kappa 0.8171), while 41 of 47 patients completed both remote and in-person follow-up; patient satisfaction was also high [15]. A multicentre REDSCAR-Trial protocol has since been published to further validate AI-based recognition of surgical wound complications in abdominal surgery [16].

Taken together, these studies suggest that remote postoperative wound assessment is feasible, but the best model in many settings may still be a simple, scalable approach rather than a highly specialised platform [17,18]. Against this background, we evaluated smartphone-based postoperative wound assessment following laparoscopic surgery in a resource-limited tertiary hospital as a supplement to routine postoperative care.

## 2. Materials and Methods

### 2.1. Study Setting and Design

This prospective cohort study was conducted over a one-year period from June 2022 to June 2023 at Surgical Unit-II, Benazir Bhutto Hospital, Rawalpindi, Pakistan, a public sector tertiary care teaching and referral hospital. Ethical approval was obtained from the institutional review board prior to study initiation. The study evaluated smartphone-based postoperative wound assessment following elective laparoscopic general surgical and bariatric procedures as a supplement to routine postoperative care.

### 2.2. Patient Recruitment

The study included adult patients aged 18 years or older who underwent elective laparoscopic surgery and were clinically stable at the time of discharge. Inclusion also required access to a smartphone with messaging capability and the ability to communicate via messaging either independently or with caregiver assistance. Patients were excluded if they required immediate postoperative inpatient care or urgent intervention at discharge, lacked access to a smartphone or internet connectivity, or were unable to use smartphone messaging because of cognitive, visual, or literacy limitations without caregiver support. Patients who declined participation were also excluded.

Consecutive sampling was used. All eligible patients undergoing elective laparoscopic procedures during the study period were approached prior to discharge and invited to participate. A total of 137 patients were assessed for eligibility. Of these, 14 were excluded because of lack of smartphone access or inability to use messaging services, and 10 declined to participate. The final study cohort consisted of 113 patients. Recruitment was continuous throughout the study period.

Written informed consent was obtained from all participants prior to discharge. Consent included participation in the study, permission for transmission of wound images via smartphone messaging, and authorization for anonymized use of clinical data for research purposes. Patients were informed regarding potential risks associated with digital communication, including privacy and confidentiality considerations.

### 2.3. Workflow

Following discharge, participants were instructed to send wound images and clinical queries to their treating surgeon within 14 days using WhatsApp Messenger (Meta Platforms, Inc., Menlo Park, CA, USA), an encrypted smartphone messaging platform. WhatsApp Messenger provided end-to-end encryption and was used solely as a communication adjunct rather than a formal medical record system. Patients received standardized instructions regarding timing of submissions, image quality requirements, including lighting, focus, and wound visibility, and avoidance of identifiable personal features.

Patients were educated before discharge on how to take clear wound photographs, and image quality was prospectively assessed during review. No images required rejection or resubmission because all submitted photographs were of acceptable quality for clinical assessment. Reminders were sent through WhatsApp Messenger when required. Fortunately, no transmission delays or failures were observed.

All wound photographs and messages were reviewed by a single consultant surgeon; therefore, interobserver agreement analysis was not applicable. The consultant surgeon provided one of three standardized responses: reassurance only, medication prescription or adjustment, or recommendation for in-person clinical evaluation.

Patients were instructed to bypass WhatsApp assessment and attend the emergency department directly if they developed severe or worsening abdominal pain, vomiting, fever, dyspnea, hemodynamic instability, wound dehiscence, heavy bleeding, purulent discharge, or any other symptom suggesting a serious postoperative complication.

### 2.4. Outcomes

The primary outcome was patient satisfaction measured using the Patient Satisfaction Questionnaire Short Form (PSQ-18), administered at the end of the 14-day follow-up period [19]. It was self-administered in either Urdu or English based on patient preference, with assistance provided when required. Although the PSQ-18 is widely used in healthcare research, no formal local validation was available for this population. Internal consistency could not be assessed in this cohort because item-level responses were not retained in a format suitable for post hoc reliability testing. Scores ranged from 18 to 90, with higher scores indicating greater patient satisfaction. Incomplete responses were excluded from the analysis.

Secondary outcomes included the proportion of patients requiring escalation to in-person review and the distribution of remote management strategies, including reassurance, medication prescription, or escalation to clinical assessment. Each patient was assigned a single primary outcome based on the highest level of intervention required, ensuring mutually exclusive categorization. Escalation was defined as recommendation for in-person evaluation due to suspected surgical complications, including surgical site infection defined according to Centers for Disease Control and Prevention criteria [20], wound dehiscence, persistent bleeding or discharge, severe pain, systemic symptoms, or surgeon clinical judgment.

False-negative analysis was not performed because the study was designed as a feasibility and satisfaction study rather than a formal diagnostic accuracy study. Remote assessment was used as an adjunct to routine care, and the study was not structured as a blinded paired validation study with a predefined reference-standard comparison across all participants.

### 2.5. Data Analysis

All patient communications via the messaging platform were monitored by the treating surgeon. Data were analysed using SPSS version 25.0. Continuous variables were expressed as mean ± standard deviation, while categorical variables were reported as frequencies and percentages. Ninety-five percent confidence intervals were calculated for key proportions. Given the exploratory and feasibility nature of the study, no formal sample size calculation was performed.

## 3. Results

**Figure 1:**
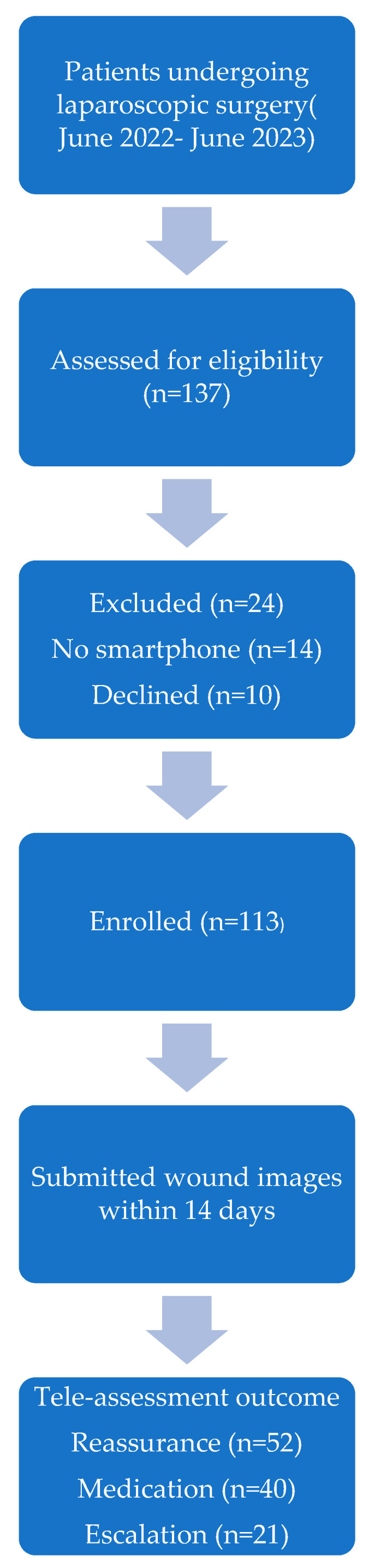
Included Patient Flow Chart.

A total of 113 patients were enrolled and all completed follow-up within the 14-day postoperative period. Figure 1 shows the patient flow chart. Reminders were sent through to 46/113 (40.7%) of the participants. Using the predefined hierarchical outcome categorization, 21 patients (18.6%) required escalation to in-person review. The remaining 92 patients (81.4%) were managed remotely, of whom 52 (46.0%) received reassurance only and 40 (35.4%) required medication prescription or adjustment. No wound images were rejected or resubmitted, as all submitted photographs were of acceptable quality for clinical assessment.

The mean PSQ-18 score for the cohort was 79.66 ± 11.24 (range 18–90), see Table 1. Patients undergoing general surgical procedures (*n* = 101) had a mean satisfaction score of 81.02 ± 12.67, compared with 75.11 ± 10.35 in the bariatric surgery group (*n* = 12). Procedure-specific mean satisfaction scores were 80.41 ± 13.29 for appendectomy, 82.29 ± 10.48 for hernia repair, 80.02 ± 10.82 for cholecystectomy, 78.12 ± 12.48 for sleeve gastrectomy, 74.44 ± 11.62 for mini gastric bypass, and 74.52 ± 8.89 for Roux-en-Y gastric bypass. Satisfaction was comparable across procedure types.

Among the 21 patients referred for in-person review, escalation occurred in appendectomy cases (*n* = 5), hernia repair (*n* = 6), and cholecystectomy (*n* = 10), with no escalations among bariatric surgery patients. The escalation reasons were recorded at the level of procedure-based triage rather than diagnosis-specific complications, and therefore a retrospective breakdown into surgical site infection, dehiscence, seroma, hematoma, or similar categories was not available. Among the 21 patients referred for in-person review, the main qualitative triggers for escalation were severe postoperative pain, suspected wound discharge, and bleeding. Although the study was not designed as a diagnostic accuracy trial, the pattern of remote reassurance, medication adjustment, and selective escalation demonstrates the practical utility of smartphone-based wound assessment as a feasible postoperative triage tool in this setting.

## 4. Discussion

Our findings suggest that smartphone-based postoperative wound assessment can function as a feasible adjunct to routine follow-up after laparoscopic surgery in a resource-limited setting. In our cohort, most postoperative concerns were managed remotely, patient satisfaction was high, and no wound images required rejection or resubmission after pre-discharge education on image quality. Taken together, these findings support a simple, patient-facing model in which a familiar messaging platform can be used to triage postoperative concerns without replacing in-person review when it is needed.

The strongest trial evidence in this area remains the TWIST study, which demonstrated that smartphone-delivered wound follow-up was feasible after emergency abdominal surgery and supported earlier diagnosis of surgical-site infection, while also improving access to care and reducing community care attendance [21]. The INROADE study extended this work into routine practice across two tertiary hospitals and showed high uptake of the intervention, strong usability and satisfaction ratings, and no major feasibility problems with the technology or interface [10]. In that study, 200 patients were enrolled, 83.0% used the tool, 16.5% developed SSI, and 72.7% of SSIs were diagnosed after discharge, reinforcing the value of remote monitoring for postoperative surveillance [22]. These studies are important because they suggest that the main benefit of digital wound follow-up may be improved triage and access rather than replacement of standard postoperative assessment [21,22,23].

Implementation studies provide further context for how such interventions are accepted in practice. Brown-Johnson and colleagues reported that a nurse-led care delivery app and telehealth system for wound care was feasible, acceptable, and clinically appropriate, with nurses and patients emphasising continuity of care, flexibility, and reduced anxiety [8]. Their findings highlight the importance of embedding digital wound care into existing clinical workflows and ensuring that staff with wound-care expertise remain directly involved [8] although quite recently chatbots are also being employed [24]. Similarly, Chen and colleagues found that a mobile app for postoperative wound care at home was highly usable and acceptable, with patients uploading wound images and receiving professional feedback; usability and acceptability were particularly high during the COVID-19 period and among rural patients, where remote care was more heavily relied upon [25]. Together, these studies suggest that technology alone is not sufficient; usability, workflow fit, and patient education are critical determinants of successful implementation.

The 2024 qualitative implementation study by Blytt et al. further supports this interpretation. Their work showed that both nurses and patients experienced remote wound telemedicine as meeting an unmet need and improving postoperative care, but they also identified barriers such as concerns about accessibility, the desire for more personalised follow-up, and the need for adequate equipment, staff time, and organisational support [26]. They also noted that implementation is easier in hospital outpatient settings than in home-based care, where individual effort and contextual constraints may be greater [26]. This is particularly relevant to our setting, where a low-cost WhatsApp-based pathway may be more realistic than a dedicated wound-monitoring platform [27,28], but where sustainability will still depend on staff organisation, patient instruction, and clear escalation pathways as it still risks underreporting [29].

Our results therefore add to the literature by showing that a simple encrypted messaging approach can be acceptable and operationally workable in a resource-limited tertiary hospital. The lack of image rejection or resubmission in our study suggests that basic pre-discharge teaching on lighting, framing, and focus can be sufficient to obtain clinically usable photographs [30]. This is a meaningful practical point for centres considering remote wound assessment but lacking the infrastructure for bespoke applications [17]. At the same time, this study should be interpreted as a feasibility evaluation rather than a diagnostic accuracy study. We therefore did not assess false negatives formally, and interobserver agreement was not applicable because all images were reviewed by a single consultant surgeon.

The key next step is to move from feasibility to validation. Future studies should assess diagnostic accuracy against a formal reference standard, quantify workload and cost implications, and evaluate scalability across different patient populations and healthcare settings. A multicentre comparative design would be particularly valuable, as it would clarify which elements of remote wound monitoring are essential and which can be simplified without compromising safety or patient experience [12]. For now, the present study supports WhatsApp-based postoperative wound assessment as a pragmatic and patient-acceptable method for selective escalation and routine postoperative support.

## 5. Conclusions

In conclusion, while challenges persist, the feasibility and relatively favourable patient satisfaction scores indicate the promising potential of wound assessment via smartphone messaging in resource-limited settings for laparoscopic procedures. Addressing challenges and tailoring interventions can pave the way for more widespread implementation, ultimately enhancing postoperative care accessibility and quality in these settings.

## 6. Limitations

This study has several limitations. First, it was a single-center feasibility study from a resource-limited setting, which may restrict generalizability to other healthcare systems. Second, wound photographs were reviewed by a single consultant surgeon; therefore, interobserver agreement and Cohen’s kappa could not be assessed. Third, the study was designed to evaluate feasibility, patient satisfaction, and selective escalation rather than formal diagnostic accuracy, so false-negative analysis against a blinded reference standard was not performed. Fourth, PSQ-18 internal consistency could not be calculated because item-level responses were not retained, and the questionnaire was not formally validated in this population especially for Urdu language translation. Finally, escalation outcomes were recorded as clinical triage decisions rather than diagnosis-specific complications, limiting detailed breakdown of the referred cases. Despite these constraints, the study provides pragmatic evidence that WhatsApp-based postoperative wound assessment may be a workable adjunct to routine surgical follow-up in settings where specialist digital platforms are unavailable.

## Figures and Tables

**Table 1:** Outcomes of smartphone-based postoperative wound assessment.

Laparoscopic Procedure	Cases	Steps Taken by Treating Surgeon			PSQ-18 Score
		Reassurance	Medication Prescribed	Patient Called For Visit (ER/OPD)	
**General Surgery Services**	**101**	**44**	**36**	**21**	**81.02 ± 12.67**
Appendectomy	31	12	14	5	80.41 ± 13.29
Hernia Repair	25	10	9	6	82.29 ± 10.48
Cholecystectomy	45	22	13	10	80.02 ± 10.82
**Bariatric Surgery** **Services**	**12**	**8**	**4**	**0**	**75.11 ± 10.35**
Sleeve Gastrectomy	3	2	1	0	78.12 ± 12.48
Mini gastric bypass	7	4	3	0	74.44 ± 11.62
Roux-en-Y Gastric bypass	2	2	0	0	74.52 ± 8.89
**Total**	**113**	**52 (46.01%)**	**40(35.4%)**	**21 (18.6%)**	**79.66 ± 11.24**

ER = Emergency Room. OPD = Outpatient Department. PSQ = Patient Satisfaction Questionnaire.

## Data Availability

The original contributions presented in the study are included in the article, further inquiries can be directed to the corresponding author.
